# Realistic simulator for craniosynostosis endoscopic approach

**DOI:** 10.3171/2021.1.FOCVID20135

**Published:** 2021-04-01

**Authors:** Giselle Coelho, Eduardo Vieira, Jose Hinojosa, Hans Delye

**Affiliations:** 1 Neurosurgery Department, Santa Marcelina Hospital, São Paulo;; 2 Scientific Department, EDUCSIM Institute, São Paulo;; 3 Neurosurgery Department, São Paulo University, USP, São Paulo;; 4 Neurosurgery Department, Sabará Children's Hospital, São Paulo, Brazil;; 5 Pediatric Neurosurgery Department, Sant Joan de Déu Barcelona Children's Hospital, Barcelona, Spain;s and; 6 Neurosurgery Department, RadboudUMC, Nijmegen, The Netherlands;

**Keywords:** craniosynostosis, surgical training, simulation, endoscopy

## Abstract

Craniosynostosis is a premature fusion of cranial sutures, and it requires surgery to decrease cranial pressure and remodel the affected areas. However, mastering these procedures requires years of supervised training. Several neurosurgical training simulators have been created to shorten the learning curve. Laboratory training is fundamental for acquiring familiarity with the necessary techniques and skills to properly handle instruments. This video presents a novel simulator for training on the endoscopic treatment for scaphocephaly and trigonocephaly, covering all aspects of the procedure, from patient positioning to performing osteotomies.

The video can be found here: https://vimeo.com/512526147.

## Transcript

**0:39 Simulation Model**. Craniosynostosis is a premature fusion of cranial sutures and requires surgery to decrease cranial pressure and remodel the affected areas. However, mastering these procedures requires years of supervised training. To shorten the training time, surgical simulation can be used. Several neurosurgical training simulators have been created to shorten the learning curve. Laboratory training is fundamental for acquiring familiarity with necessary techniques and skills to properly handle instruments.^1,2^ The main goal of this video is to present a novel simulator for the training of the endoscopic treatment for scaphocephaly and trigonocephaly, covering all aspects of the procedure, from patient positioning to performing osteotomies. The head model is based on a combination of real CT images obtained from a scaphocephaly patient and a trigonocephaly patient. From the DICOM images, a three-dimensional reconstruction was created of a skull shape with both scaphocephalic and trigonocephalic features. A special resin in the shape of the skull constituted the basic structure of the craniosynostosis training module. This was complemented with a synthetic reproduction of brain tissue and the superior sagittal sinus. The training module is then connected to a puppet infant body by a simulated cervical spine (acrylic composition). In this way, the head is locked by a simple acrylic connection. Using this model, training is performed as if it was a real operation on a real infant. The model is commercially available and can be used for training on hands-on courses and in regular neurosurgical residency curriculum.

### 2:36 Scaphocephaly Approach

**2:40 Demonstration of Placement and Skin Incision**. For scaphocephaly, the model is placed in sphynx position, taking care that the head is placed as extended as possible, parallel to the floor. For training purposes, the areas of the intended bone resection are being drawn on the skin. Next, the skin incisions are drawn, one behind the anterior fontanelle and one before the posterior fontanelle.^3–5^

**3:14 Endoscopic Technique**. The first step is the dissection of skin and galea from the bone. This phase can be done with a direct vision or with rigid endoscopic assistance. The second step should be the dissection of dura from the bone. The endoscope is used to take out some emissary veins along the way that can be coagulated with a bipolar forceps.^3–5^ This step may reduce the blood loss. This is an endoscopic view of the model showing the superior sagittal sinus just below the sagittal suture. The scaphocephaly model has a real good representation of this sinus.^1,2^ Trainees can try to push down the dura and take out the overlying bone.

### 4:06 Metopic Approach

**4:09 Demonstration of Placement and Skin Incision**. The model is placed in supine position. The skin incision is drawn as low as possible just behind the hair line, which is sometimes difficult to establish in small babies. A W-shaped skin incision will allow to progress a safe subgaleal dissection and bone resection.^3–5^ The model presents skin consistency quite similar to human skin, allowing trainees to experience the difficulties of the technique.^1,2^

**4:48 Endoscopic Technique**. In the first step, skin is dissected from the bone. Then, the trainee can enter the endoscope to perform epidural dissection and bone removal. It is very important that the bone resection is progressively following the contour of the bone to avoid too much dura and brain compression. Rongeurs, heavy bone scissors, and chisels can be used to perform the osteotomy up to the frontonasal suture.^1–6^

## Author Contributions

Primary surgeon: Coelho, Hinojosa. Assistant surgeon: Delye. Editing and drafting the video and abstract: Coelho, Vieira, Delye. Critically revising the work: all authors. Reviewed submitted version of the work: all authors. Approved the final version of the work on behalf of all authors: Coelho. Supervision: Coelho. Defining video content/format together with corresponding author: Delye.
